# Bladder cancer incidence and mortality among men with and without castration therapy for prostate cancer – a nation-wide cohort study

**DOI:** 10.2340/1651-226X.2024.40969

**Published:** 2024-09-25

**Authors:** Josephine M. Hyldgaard, Mette Nørgaard, Peter E. Hjort, Jørgen B. Jensen

**Affiliations:** a Deparment of Clinical Medicine, Aarhus University, Aarhus, Denmark; b Department of Urology, Aarhus University Hospital, Aarhus, Denmark; c Department of Clinical Epidemiology, Aarhus University Hospital, Aarhus, Denmark

**Keywords:** Urinary bladder cancer, antihormonal treatment, androgen receptor, gender

## Abstract

**Background and purpose:**

Bladder cancer (BC) is a common malignancy in the Western World with men being diagnosed almost four times as often as women. The etiology of bladder cancer may involve sex hormones. Prostate cancer (PCa) patients treated with chemical castration, such as androgen deprivation therapy, or surgical castration, may therefore have a lower risk of developing bladder cancer.

**Patients/material and methods:**

In a nation-wide population-based cohort study using national Danish registry data, we included a cohort of men with a first-time PCa diagnosis between 2002 and 2018 divided according to antihormonal treatment in the first year after PCa diagnosis and a comparison cohort consisting of 10 age-matched persons for each PCa patient. Each individual was followed from 1 year after PCa diagnosis until death or end of follow-up. We computed cumulative incidences (risk) and hazard ratios (HRs) for BC. In a second cohort analysis, we determined overall survival and BC-specific mortality, determined from date of BC diagnosis until death.

**Results and interpretation:**

We included 48,776 PCa patients of whom 13,592 were treated with chemical castration, 2,261 with surgical castration, and 32,923 received no antihormonal treatment. The 5-year risk of BC for each PCa group was 1.1%, 0.7%, and 1.3%, respectively, corresponding to an adjusted HR of 1.13 (95% CI 0.98; 1.31), 0.95 (95% CI 0.62; 1.47), and 1.18 (95% CI 1.09; 1.28) compared to individuals without PCa. Patients receiving antihormonal treatment had a slightly lower incidence of BC compared to individuals without PCa, however, this was not supported by the HRs. The treatment, however, was not associated with overall survival.

## Introduction

Bladder cancer (BC) is the most prevalent urinary tract tumors among men in the Western world. Well-known risk factors of BC include age, occupational exposure, and smoking, where smoking accounts for approximately 50% of all incident bladder tumors [1]. Another risk factor is male sex with and incidence rate ratio of 4:1 for men compared to women [2, 3]. When adjusting for exposures such as cigarette smoking, occupational hazards or urinary tract infections, the risk difference between the sexes remains [4]. Thus, sex-hormone signaling, through the interaction between testosterone and androgen receptors (AR), may be a contributor to the development of BC [5]. Sex-hormone signaling in cancers is predominantly known from breast and prostate cancer (PCa). In the management of PCa, antihormonal treatment, also referred to as endocrine therapy, includes the following groups castration therapy (surgical or chemical), anti-androgens, 5-alpha reductase inhibitors, and androgen synthesis inhibitors. Chemical castration consists of androgen deprivation therapy (ADT) or surgical castration and plays a significant role, by inhibiting the growth of cancer cells. ADT comprises GnRH-antagonists or GnRH-agonists, which block the androgen pathway. Surgical castration renders a similar decrease in the testosterone level [6]. Anti-androgens are competitive inhibitors of the binding of androgens to their receptors. This treatment, however, is rarely used as monotherapy for PCa [7]. As sex hormones have the potential to be pro-oncogenic and potentially also increase the risk of BC in the urothelium, antihormonal treatment may be anti-oncogenic [8]. In a review of observational studies, Santella et al. reported five studies in which ADT showed a protective effect on BC with hazard ratios ranging from 0.29 to 0.93. One study showed no association, and one reported an increased risk of BC in users of ADT [9]. Three studies reported an association between ADT and reduced BC recurrence [10–12], and three studies suggested a potential association between ADT and reduced incident BC [13–15]. The interpretation of these results, however, was hampered due to potential biases (immortal time bias [13, 14] and potential residual confounding [13–15]). Thus, the association between antihormonal treatment and risk of BC remains uncertain and in need of continuous research, as it may yield potential therapeutic targets or treatment modifiers in BC. Inspired by this hypothesis, the objective of this cohort study was to examine whether there is a lower incidence of BC or difference in BC prognosis in men undergoing antihormonal treatment for PCa, by either chemical or surgical castration, compared with men with PC and no antihormonal treatment and men without PC.

## Patients/materials and methods

### Study design and setting

We conducted a nation-wide population-based cohort study. The primary analysis assessed the risk of BC among men with and without PCa and antihormonal treatment. In a secondary analysis we assessed mortality among those who had a BC diagnosis according to their PCa and antihormonal treatment status. The study was conducted in Denmark, which has a population of approximately 5.9 million inhabitants (2024). All residents have equal free access to tax-supported health care [16]. Furthermore, since 1968, every newborn or immigrant has been assigned a unique registration number from the Civil Registration System (CRS), which allows for unambiguous data linkage for each individual [17]. This setting allows for a large cohort with detailed information regarding each patient’s medical history.

### Participants

We used the Danish National Patient Register (DNPR) to identify all patients diagnosed with first-time PCa, without any previous cancer diagnosis except nonmelanoma skin cancer, from 2002 to 2018. DNPR contains information regarding all patient admissions to Danish hospitals since 1977 and all admissions to outpatient clinics since 1995. Diagnoses are recorded according to the International Classification of Diseases, version 10 (ICD-10) [18].

**Figure 1:**
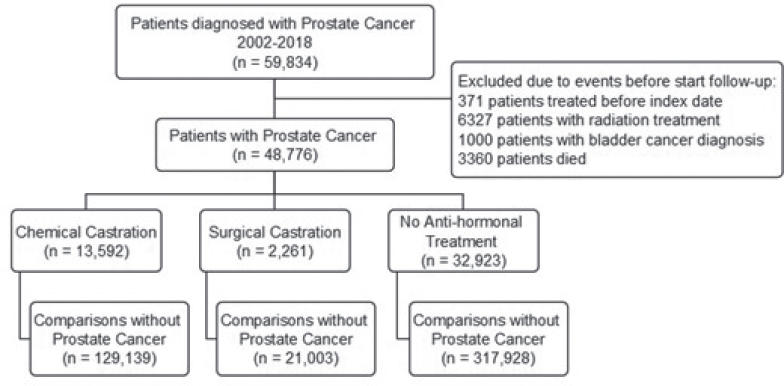
Flowchart of patient subgroups.

The CRS, which is an administrative register established in 1968, containing individual-level information, such as migration and vital status, on all Danish residents [17], was used to identified a comparison cohort consisting of 10 age and sex-matched persons for each PCa patient. Index date was registered as the date of the patient’s initial PCa diagnosis, and the corresponding date for men in the comparison groups. Patients were followed from 1 year after index date until date of BC, death, or end of follow-up (December 31^st^, 2021), whichever occurred first. Patients with radiation treatment before 1 year were excluded due to their increased risk of secondary pelvic cancer, regardless of adjuvant ADT (Figure 1).

### Variable definition

#### Treatment

DNPR includes information regarding diagnosis, treatment, and procedures related to PCa from before and after any potential multidisciplinary team discussion. Based on this information, we categorized patients into exposure subgroups depending on their PCa treatment during the first year after PCa diagnosis as either chemical castration, surgical castration, or no antihormonal treatment. Patients who had both chemical and surgical castration were categorized as surgical castration, as this treatment is considered to have permanent effect on hormonal status. We ignored treatment after the first year. Chemical castration only included ADT treatment with either GnRH antagonists or GnRH agonists, as information on per-oral anti-androgen treatment such as bicalutamide was not consistently registered in the DNPR and in-hospital medication is not included in the Danish National Prescription Register [19].

#### Bladder cancer

In the primary analysis, the outcome of interest was BC recorded in the National Pathology Register (see supplementary Table S1 for codes). This register contains information regarding all pathologic procedures and diagnoses from patients in the Danish National Health Care system since 1995 [20]. We included both nonmuscle invasive and muscle invasive bladder tumors. Date of BC diagnosis was registered as the first entry in the register. Patients diagnosed with BC within the first year of their PCa diagnosis were excluded as these cancers were less likely to be related to PCa treatment. BC stage at diagnosis was categorized into nonmuscle invasive bladder cancer (NMIBC), muscle invasive bladder cancer (MIBC), and unknown bladder cancer grade (pTx).

#### Survival and BC-specific mortality

Overall survival (OS) and BC-specific mortality were determined among patients who were diagnosed with BC during the study period, regardless of PCa status and antihormonal treatment. Follow-up for both OS- and BC-specific mortality started at date of BC. The Cause of Death Register was used to determine time and cause of death. The Cause of Death Register is based on the certificate of death completed by a physician and has had computerized records since 1970. The register contains information on cause of death but also on time and place of death [21]. OS were presented in Kaplan-Meier survival plots and BC-specific mortality was presented as cumulative incidences in Aalen-Johansen curves, with the competing risk of death from other causes. For each group, 1-year survival estimates and 1-year BC-specific mortalities were determined from these curves. Cox regression was used to compute an adjusted HR of death (overall and BC-specific death). The HR was adjusted for age, heart disease, vascular disease, and chronic obstructive pulmonary disorder.

#### Covariates and confounders

Comorbidity is associated with an increased mortality risk. Comorbidities were defined according to the Charlson Comorbidity Index (CCI) and were computed based on information from the DNPR. We categorized comorbidity based on the CCI-score into 0, 1–2, >2. We also specifically included smoking-related comorbidities, defined as chronic obstructive pulmonary disorder, heart disease, or vascular disease, such as atherosclerosis in the analysis to, at least partly, account for the confounding effect of smoking on risk of BC. Other drugs, such as 5-alpha reductase inhibitors exert their effect in prostatic tissue, converting testosterone to the more potent dihydrotestosterone. Information regarding this treatment was retrieved from the National Danish Prescription Register [19].

### Statistical analyses

Descriptive results of patients with and without PCa were reported using median, 25^th^ and 75^th^ percentiles for continuous variables. For categorial variables, we reported frequencies and proportions.

#### Cumulative incidences using landmark analysis

To compare cumulative incidences, we used a landmark approach starting follow-up one year after index date. Patients who died or had a BC before the landmark were excluded. We censored cohort members at BC, death, emigration, and end of study period, whichever occurred first. If men in the comparison cohorts were diagnosed with PCa, they were censored from further follow-up in the comparison cohort and moved to the PCa cohort.

We plotted cumulative incidence of BC using Aalen-Johansen curves with death as a competing risk and estimated 5- and 10-year risks of BC. We compared BC incidence as crude and adjusted hazard ratios (HR) using Cox-regression analysis, adjusting for age and smoking-related-comorbidities.

#### Sensitivity analysis

As a sensitivity analysis, we changed the landmark from 1 to 2 years after index date.

#### Overall survival and BC-specific mortality

Survival analysis was performed on patients diagnosed with BC within the study period. We compared BC patients with prior PCa according to their antihormonal treatment, to BC patients without PCa. Overall survival (OS) was presented as Kaplan-Meier survival plots and 1-year survival estimates. For BC-specific mortality, we presented Aalen-Johansen curves with death from other causes as competing risk, and we estimated 1-year disease-specific mortality risk. For both survival and mortality estimates, we used Cox regression analysis to estimate adjusted hazard ratios.

All analyses were done in R Studio version 4.4.2 (Boston Massachusetts, USA) [22]. The study was reported to the Danish Data Protection Agency (Central Denmark Region, record no. 1-16-02-128-20) and adheres to the General Data Protection Regulations. According to Danish regulations, the study did not require separate ethical approval as it was based on routinely collected registry data.

## Results

We identified 48,776 PCa patients of whom 15,853 received antihormonal treatment within the first year of PCa diagnosis. Of these, 13,592 patients were chemically castrated and 2,261 surgically castrated. The remaining 32,923 PCa patients did not receive antihormonal treatment. The PCa patients were matched with 129,139, 21,003, and 317,928 respective men without PCa, as comparison cohorts (Figure 1). The distribution of comorbidities was similar between the groups. The use of 5-alpha reductase inhibitors was similar for patients with PCa, and marginally lower for patients without PCa. Average follow-up time for patients with PCa was 50.4 months, compared to 75.2 months for patients without PCa (Table 1). This difference was particularly pronounced for patients with chemical or surgical castration.

**Table 1:** Descriptive analysis.

	Chemical Castration and men in the comparison group		Surgical Castration and men in the comparison group		No Antihormonal Treatment and men in the comparison group	
	PCa (*n* = 13,592)	No PCa (*n* = 129,139)	PCa (*n* = 2,261)	No PCa (*n* = 21,003)	PCa (*n* = 32,923)	No PCa (*n* = 317,928)
Age, median (p25, p75)	73 (67, 79)	73 (67, 79)	77 (70, 82)	76 (69, 82)	68 (63, 74)	68 (63, 73)
Follow-up time (years), median (p25, p75)	3.7 (1.9, 6.4)	5.8 (3.3, 9.4)	2.5 (0.9, 5.1)	6.1 (3.5, 9.5)	6.4 (3.6, 10.1)	6.9 (3.9, 10.7)
Prostatectomy, *n* (%)	437 (3)	*NA*	13 (1)	*NA*	10,376 (32)	*NA*
Charlson Comorbidity Index, *n* (%)						
0	8,635 (64)	79,441 (62)	1,345 (59)	12,702 (60)	23,219 (71)	216,911 (68)
1–2	4,437 (33)	43,918 (34)	807 (36)	7,376 (35)	8,936 (27)	90,677 (29)
> 2	520 (4)	5,780 (5)	109 (5)	925 (4)	768 (2)	10,340 (3)
Prostate Cancer						
Clinical T-stage, *n* (%)						
T1	1,469 (11)	*NA*	244 (11)	*NA*	12,105 (37)	*NA*
T2	2,558 (19)	*NA*	259 (11)	*NA*	9,013 (27)	*NA*
T3	5,531 (41)	*NA*	809 (36)	*NA*	3,832 (12)	*NA*
T4	875 (6)	*NA*	251 (11)	*NA*	213 (0.6)	*NA*
Tx	1,673 (12)	*NA*	340 (15)	*NA*	2,733 (8.1)	*NA*
NA	1,486 (11)	*NA*	358 (16)	*NA*	5,027 (15.3)	*NA*
Clinical N-stage, *n* (%)						
N0	1,713 (13)	*NA*	116 (5)	*NA*	7,332 (22)	*NA*
N1	1,714 (13)	*NA*	245 (11)	*NA*	722 (2)	*NA*
Nx	< 5 (0)	*NA*	< 5 (0)	*NA*	< 5 (1)	*NA*
NA	10,165 (74)	*NA*	1,900 (84)	*NA*	24,867 (76)	*NA*
Clinical M-stage, *n* (%)						
M0	3,830 (28)	*NA*	257 (11)	*NA*	12,494 (38)	*NA*
M1	3,575 (26)	*NA*	869 (38)	*NA*	880 (3)	*NA*
Mx	4,701 (35)	*NA*	777 (34)	*NA*	14,522 (44)	*NA*
NA	1,486 (11)	*NA*	358 (17)	*NA*	5,027 (15)	*NA*
Smoking-related comorbidities, *n* (%)						
Ischemic heart disease	2,121 (16)	21,678 (17)	389 (17)	3,541 (17)	4,202 (13)	43,156 (14)
COPD	537 (4)	5,201 (4)	92 (4)	891 (4)	920 (3)	10,108 (3)
Vascular disease	412 (5)	7,589 (6)	155 (7)	1,289 (6)	1,183 (4)	14,160 (5)
5-alpha reductase inhibitor users, *n* (%)	1,558 (11)	10,701 (8)	281 (12)	1,976 (9)	3,571 (11)	17,330 (6)

PCa: Prostate Cancer; p25: 25^th^ percentile; p75: 75^th^ percentile, COPD: chronic obstructive pulmonary disorder; NA: not available.

### Bladder cancer

**Figure 2:**
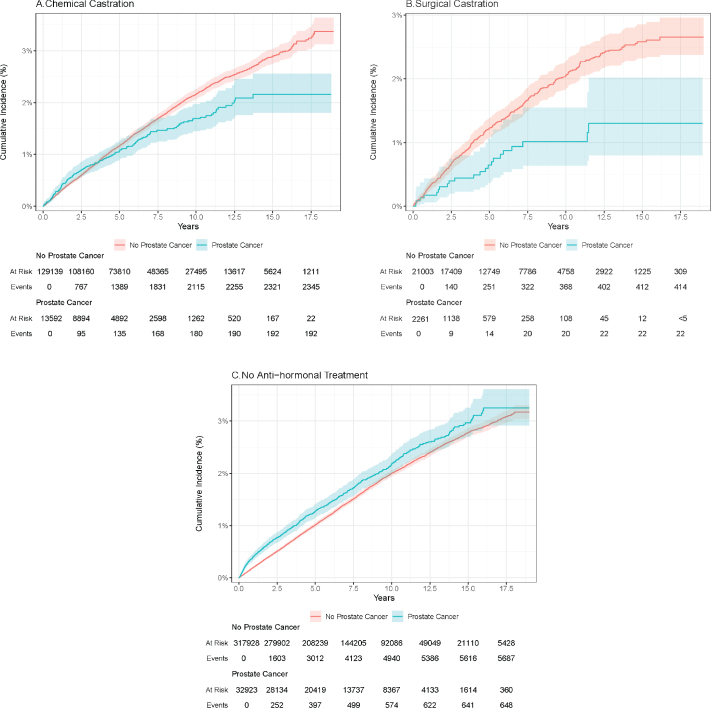
Cumulative incidences of bladder cancer (%). (A) Chemical castration. (B) Surgical castration. (C) No antihormonal treatment.

During follow-up, we identified a total of 9,318 incident BC cases (Table 2). The 5-year risks of BC were 1.1% (95% CI: 0.9; 1.3) for chemically castrated patients and 0.7% (95% CI: 0.4; 1.1) for surgically castrated patients and 1.3% (95% CI: 1.2; 1.4) for patients with PCa but no antihormonal treatment. The 5-year risk of BC was lower for patients with antihormonal treatment compared to individuals without PCa (Figure 2A, B), and the 5-year risk of BC was higher for patients with PCa who did not receive any antihormonal treatment in the first year compared to individuals without PCa (Figure 2C).

**Table 2:** Incident Bladder Cancer.

	Chemical Castrationand men in the comparison group		Surgical Castration and men in the comparison group		No Antihormonal Treatment and men in the comparison group	
	PCa (*n* = 192)	No PCa (*n* = 2,348)	PCa (*n* = 22)	No PCa (*n* = 414)	PCa (*n* = 648)	No PCa (*n* = 5,694)
Bladder Cancer stage, *n* (%)						
NMIBC	128 (66)	1.664 (71)	15 (68)	271 (65)	397 (61)	4,036 (71)
MIBC	64 (34)	679 (29)	7 (32)	141 (34)	250 (39)	1,644 (29)
pTx	< 5 (0)	5 (0)	< 5 (0)	< 5 (1)	< 5 (0)	14 (0)
Charlson Comorbidity Index, *n* (%)						
0	129 (68)	1,427 (61)	12 (55)	264 (64)	464 (71)	3,777 (66)
1–2	56 (29)	821 (35)	8 (36)	138 (33)	174 (27)	1,776 (31)
> 2	7 (4)	100 (4)	2 (9)	12 (3)	10 (2)	141 (3)
Risk of Bladder Cancer, % [95% CI]						
5-year	1.1 [0.9, 1.3]	1.2 [1.1, 1.2]	0.7 [0.4, 1.1]	1.2 [1.1, 1.4]	1.3 [1.2, 1.4]	1.0 [1.0, 1.0]
10-year	1.7 [1.5, 2.0]	2.2 [2.1, 2.3]	1.0 [0.6, 1.5]	2.1 [1.8, 2.3]	2.2 [2.0, 2.4]	2.0 [1.0, 2.1]
HR for Bladder Cancer [95% CI]						
Crude HR	1.14 [0.99, 1.32]	Ref	0.98 [0.63, 1.51]	Ref	1.18 [1.08, 1.28]	Ref
Adjusted HR	1.13 [0.98, 1.31]	Ref	0.95 [0.62, 1.47]	Ref	1.18 [1.09, 1.28]	Ref

PCa: Prostate Cancer; NMIBC: Non-muscle invasive bladder cancer; MIBC: Muscle invasive bladder cancer; pTx: Tumor of unknown location; HR: Hazard ratio.

Compared with matched individuals without PCa, the crude HR of BC was 1.14 (95% CI: 0.99; 1.32), 0.98 (95% CI: 0.63; 1.51), and 1.18 (95% CI: 1.08; 1.28) for patients with PCa and chemical castration, surgical castration, and no antihormonal treatment, respectively. After adjustment, the HR was 1.13 (95% CI: 0.98; 1.31) for patients with chemical castration. Adjusted HR for patients with surgical castration and no antihormonal treatment were 0.95 (95% CI: 0.62; 1.47) and 1.18 (95% CI: 1.09; 1.28), respectively (Table 2).

Results rendered no clear difference in BC staging at time of diagnosis although patients with chemical castration and patients with no antihormonal treatment for PCa were slightly more often diagnosed with MIBC compared to their respective comparison cohorts.

When setting the landmark 2 years after index date, the 5-year risks of BC were 1.1% (95% CI 0.91; 1.3), 0.83% (95% CI 0.5; 1.3), and 1.1% (0.96; 1.2), for patients with chemical castration, surgical castration, and no antihormonal treatment, respectively. The corresponding adjusted HRs were 1.12 (95% CI 0.96; 1.31), 0.98 (95% CI 0.63; 1.53), and 1.06 (95% CI 0.97; 1.17).

### Overall survival and BC-specific mortality

**Figure 3:**
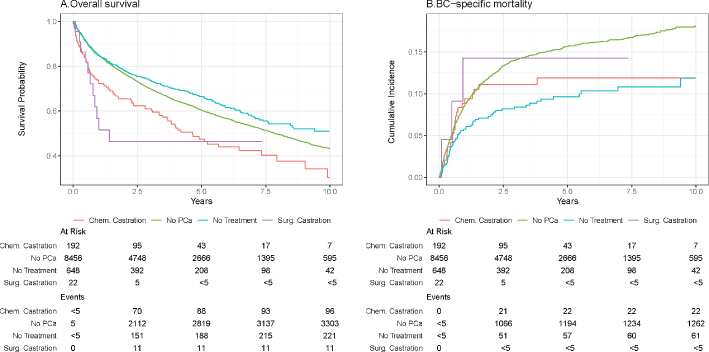
Survival and prognosis following BC diagnosis. (A) Overall survival. (B) BC-specific mortality.

A total of 9,318 patients, regardless of exposure, were diagnosed with incident BC during follow-up time. The 1-year overall survival estimates of patients with PCa and chemical castration, surgical castration, or no antihormonal treatment were 73%, 57%, and 85%, respectively, and for individuals without PCa, it was 85% (Figure 3A). The adjusted HR for overall survival suggested higher mortality for patients with antihormonal treatment compared to patients without antihormonal treatment as well as individuals without PCa (Table 3). The 1-year BC-specific mortality was 9.5%, 14%, 5.8% for patients with chemical castration, surgical castration, and no antihormonal treatment, respectively. For individuals, without PCa the 1-year BC-specific mortality was 8.6% (Figure 3B). BC-specific HR was lower for patients with chemically castration as well as for patients without antihormonal treatment, compared to individuals without PCa (Table 3).

**Table 3:** Crude and adjusted HR for overall survival and BC-specific mortality.

	Chemical castration	Surgical castration	No antihormonal treatment	No Prostate cancer
Adjusted HR OS	1.45 [1.16, 1.81]	1.50 [0.78, 2.89]	1.05 [0.92, 1.20]	*Ref*
Crude HR for OS	1.47 [1.18, 1.82]	2.04 [1.03, 4.02]	0.87 [0.76, 0.99]	*Ref*
OS, % [95% CI]				
1-year	73 [67, 79]	57 [39, 83]	85 [82, 88]	85 [84, 86]
5-year	47 [40, 56]	46 [29, 74]	67 [63; 71]	60 [59; 62]
Adjusted HR for BC-specific mortality	0.84 [0.55, 1.27]	0.97 (0.31, 3.02]	0.74 [0.58, 0.96]	*Ref*
Crude HR for BC-specific mortality	0.86 [0.56, 1.31]	1.26 [0.41, 3.92]	0.64 [0.50, 0.82]	*Ref*
BC-specific mortality, % [95% CI]				
1-year	9.5 [5.8, 14.0]	14.0 [3.3, 33.0]	5.8 [4.1, 7.7]	8.6 [8.0, 9.2]
5-year	12.0 [7.7, 17.0]	14.0 [3.3, 33.0]	9.6 [7.4, 12.0]	16.0 [15.0, 16.0]

HR: Hazard ratios; OS: overall survival; Ref: reference.

## Discussion

In this population-based cohort study, we found a lower 5-year risk of incident BC for patients with PCa and either chemical or surgical castration compared to individuals without PCa. In contrast, we observed a higher 5-year risk of BC for patients with PCa but no antihormonal treatment. The adjusted HRs of incident BC, however, were higher for both chemical castrated patients and patients without antihormonal treatment, when compared to individuals without PCa. Thus, we observed no clear effect of antihormonal treatment on BC incidence.

To investigate the effect of antihormonal treatment on BC prognosis, we reported overall survival and BC-specific mortality. Contrary to our hypothesis, we found a lower overall survival for patients receiving antihormonal treatment. Furthermore, the BC-specific mortality was similar regardless of treatment and higher compared to patients without antihormonal treatment. Accordingly, the adjusted HR for BC-specific death was substantially lower for patients with no antihormonal treatment compared to individuals without PCa. For patients with either chemical or surgical castration, the adjusted HR for BC-specific death was slightly lower, which may be suggestive of a somewhat protective effect of antihormonal treatment on the BC-specific outcome. However, these differences may also be due to a confounding effect of age and comorbidities; thus antihormonal treatment does not seem to influence the prognosis of BC.

Our findings on risk of incident BC are similar to Moschini et al. who divided patients according to ADT status and found a 10-year risk of BC for ADT users of 1.75% compared to 1.99% in nonusers [14]. We extended these findings by presenting lower BC risks for patients with surgical castration as well.

Shiota et al. compared incident BC in patients with PCa and treatment with either radiation therapy, prostatectomy, or primary ADT. During follow-up, BC occurred in 0% of patients with ADT, compared to 2.2% of patients with radiation therapy and 1.1% of patients with prostatectomy [13]. Based on these findings, and the effect of radiation on risk of secondary cancers, we chose to exclude patients with radiation therapy in our analyses.

Hormone status have been suggested to be involved in BC grade and androgen receptor (AR) mRNA been shown to be significantly increased in NMIBC compared to MIBC [23]. In this study, we did not, however, observe any substantial differences in BC stage at time of diagnosis.

By stratifying according to castration type, we were able to include information regarding each treatment modality, where surgical castration renders a permanent testosterone decrease, regardless of follow-up time. This group presented with a lowered risk of incident BC when compared to individuals without PCa. This was, however, likely explained by a higher risk of death before having BC due to a higher median age, more severe PCa T-stage, as well as a higher proportion of metastatic disease, compared to the other groups. Our adjusted HR could not confirm a higher incidence of BC for these patients. To our knowledge, this is one of the first studies to report risk of BC by separate castration treatment groups

Findings from molecular-based animal research have indicated sex hormones, such as testosterone, and AR play a role in bladder carcinogenesis and BC progression. When chemically inducing BC, using N-butyl-N-(4-hydroxybutyl) nitrosamine (BBN), wildtype male mice were shown to develop more BC than castrated male mice [24, 25]. Furthermore, synthetic estrogen has been shown to inhibit bladder carcinogenesis, whereas testosterone stimulated tumor growth [26–28].

Similar tendencies have been observed in in vivo studies with humane cancer cell lines. Treatment with dihydrotestosterone (DHT) significantly increased the AR expression in cells and activated a signaling pathway, known to be of significance in cancer progression [29]. Based on these results, AR signaling has been suggested as a potential therapeutic target in BC. In the present study, we aimed to provide clinical evidence of difference in BC incidence based on antihormonal treatment.

## Strengths and limitations

By using Denmark’s unique registration system, we were able to conduct a large cohort study with a virtually complete and long follow-up. We were furthermore able to include a large reference group for each study population. Additionally, we could include death by other causes as a competing event in our analyses. To address the potential detection bias caused by likely higher BC diagnostic activity through cystoscopy in men being followed in a urological outpatient clinic, with a presumably higher BC diagnostic activity through cystoscopy, we included a comparison of PCa patients with no antihormonal treatment as we expect these men were also seen regularly in an urological outpatient clinic. This is also true for the remaining groups of PCa patients; thus the impact of antihormonal treatment on BC incidence may be underestimated.

A limitation of this study, however, could be the potential detection bias caused by a lower diagnostic BC activity among patients with chemical castration. Many of these patients have advanced PCa, and if they had hematuria this could be attributed to the PCa, rather than lead to suspicion of BC. Furthermore, some patients with ADT may have received palliative care for metastatic disease and would therefore not receive further diagnostic evaluation in case of signs of a second malignancy. This bias would lead to an underestimation of BC incidence in the chemical castration group and may contribute to the lower 5-year risk of incident BC we observed.

Our study has several other limitations that should be considered. We lacked information about smoking, a major risk factor for BC. Even though we included smoking-related comorbidities to account for this, we may have residual confounding. Still, the included comorbidities were equally distributed among our exposure groups speaking against major residual confounding due to smoking. Furthermore, as we used the landmark approach, we did not include information on ADT initiated later than one year of PCa diagnosis. However, when we changed the landmark from 1 year after PCa to two years after, the adjusted HR remained virtually unchanged, which speaks against major bias due to misclassification of exposure. We did not include data on BC recurrence in this analysis, nor did we include information on patient’s treatment for BC. We also lacked information regarding lower urinary tract symptoms (LUTS), which many patients with PCa will experience to some degree. LUTS patients have an increased risk of urinary tract infections, which are associated with an increased risk of BC [30]; however, LUTS is likely as prevalent among men in the comparison groups as well.

Furthermore, with increasing age, men are more likely to be diagnosed with benign prostate hyperplasia and are prescribed 5-alpha reductase inhibitors. This treatment affects the potency of testosterone and does not directly affect the levels of testosterone, as androgen deprivation therapy drugs. Assumably, this treatment would not influence a potential effect of antihormonal treatment on the risk of BC. Furthermore, in this study, treatment with 5-alpha reductase inhibitors was equally distributed among patients with PCa as well as among men in the comparison groups and did not affect the HR estimates of BC incidence.

Another limitation of this study is the lack of information on per-oral antiandrogen treatment, which is not included in the DNPR. Accordingly, we cannot entirely rule out that some of the PCa patients classified as no antihormonal treatment had in fact received per-oral anti-androgen treatment, which could bias our results. Monotherapy with anti-androgens for PCa is, however, rarely used; thus most of these patients would presumably also receive ADT at some point and we therefore expect the potential bias to be minor.

To avoid immortal time bias, we analyzed data using a landmark approach. As a consequence of this design, PCa patients who started antihormonal treatment after the first year of PCa diagnosis would be incorrectly classified as nonusers that could lead to bias towards no effect.

We included a sensitivity analysis with a landmark after two years, which showed no changes in BC incidence.

Additionally, we censored patients at death; thus a higher mortality in patients with PCa would therefore not per se lead to lower incidence per time unit. However, it is possible that those who died were the most susceptible for BC, and the resulting selection bias (depletion of susceptible) could lead to a lower BC incidence.

In conclusion, in this national cohort study, we could not confirm our hypothesis that antihormonal treatment of PCa was followed by a lowered risk of BC. We did find that BC incidence was lower in PCa patients undergoing chemical or surgical castration compared to individuals without PCa, but this was most likely explained by a higher risk of dying before having a BC diagnosis. We found a higher risk of incident BC among patients with PCa not undergoing antihormonal treatment compared to individuals without PCa. We did not find any significant effect of either chemical or surgical castration on overall survival following BC, but we did observe a slightly lower HR of BC-specific death although this was true for all patients with PCa when compared to individuals without PCa. Thus, antihormonal treatment most likely does not affect BC prognosis in patients with both PCa and BC.

## Supplementary Material

Bladder cancer incidence and mortality among men with and without castration therapy for prostate cancer – a nation-wide cohort study

## Data Availability

Data scripts available upon contacting corresponding author JH. No access to raw data available as this is kept in government registers.
