# Supplementary material for: Bladder cancer incidence and mortality among men with and without castration therapy for prostate cancer – a nation-wide cohort study
Source: Acta Oncol. 2024 Sep 25;63:40969. doi: 10.2340/1651-226X.2024.40969 (PMC11445587; doi:10.2340/1651-226X.2024.40969)
Supplement: Bladder cancer incidence and mortality among men with and without castration therapy for prostate cancer – a nation-wide cohort study [file AO-63-40969-s1.pdf]

Supplementary material has been published as submitted. It has not been copyedited, or typeset by Acta Oncologica

Supplementary Material

Table S1. Codes used in the study.

	DNPR (2002-2021) ICD-10	Danish National Pathology Registry
Prostate Cancer	DC61*	
Non-melanoma skin cancer	DC44*	
**Prostate Cancer Treatment**		
Radiotherapy	BWG*	
Prostatectomy	KKEC*	
Androgen Deprivation Therapy	BWHC3*, BWHC5*	
Surgical Castration	KKFC10, KKFC11, KKFC13	
**Cancer Outcomes**		
Urinary Bladder		T74* with either M81202*, M81203*, M81302*, M81303* or ÆF18*
**Smoking-related comorbidities**		
Heart disease/angina pectoris	DI20*-DI25*	
Chronic Obstructive Pulmonary Disorder	DJ43*, DJ44*	
Vascular disorders/atherosclerosis	DI70*, DI71*	

ICD indicates International Classification of Diseases; DNPR, Danish National Patient Registry

Table S2. International Classification of Diseases (ICD) codes used for the conditions included in the Charlson Comorbidity Index

	Disease	ICD-10	Score
1	Myocardial infarction	I20-I25	1
2	Congestive heart failure	I50, I11.0, I13.0, I13.2	1
3	Peripheral vascular disease	I70-I71	1
4	Cerebrovascular disease	I60–I69, G45, G46	1
5	Dementia	F00–F03, F05.1, G30	1
6	Chronic pulmonary disease	J43-J44	1
7	Connective tissue disease	M05, M06, M08, M09, M30, M31, M32, M33, M34, M35, M36, D86	1
8	Ulcer disease	K22.1, K25–K28	1
9	Mild liver disease	B18, K70.0–K70.3, K70.9, K71, K73, K74, K76.0	1
10	Diabetes	E10.0, E10.1, E10.9<br>E11.0, E11.1, E11.9	1
11	Hemiplegia	G81-G82	2
12	Moderate to severe renal disease	I12-I13, N00–N05, N07, N11, N14, N17–N19, Q61	2
13	Diabetes with end-organ failure	E10.2–E10.8<br>E11.2–E11.8	2
14	Any tumor (excluded)	C00–C75	2
15	Leukemia (excluded)	C91–C95	2
16	Lymphoma (excluded)	C81–C85, C88, C90, C96	2
17	Moderate to severe liver disease	B15.0, B16.0, B16.2, B19.0, K70.4, K72, K76.6, I85	3
18	Metastatic solid tumor (excluded)	C76-C80	6
19	AIDS		6
